# Binary phase-only gallium oxide diffractive optical element for beam shaping

**DOI:** 10.1038/s41598-025-89663-0

**Published:** 2025-02-11

**Authors:** Wei Jia, Steve Blair, Berardi Sensale-Rodriguez

**Affiliations:** Department of Electrical and Computer Engineering, The University of Utah, Salt Lake City, UT 84112 USA

**Keywords:** Binary phase, Gallium oxide, Diffractive optical element, Beam shaping, Optics and photonics, Optical materials and structures

## Abstract

This study presents an experimentally validated demonstration of an inverse-optimized binary phase-only gallium oxide diffractive optical element (DOE). This DOE transforms an incident Gaussian beam into a square flat-top beam at the working plane. The design methodology for this binary phase-only DOE beam shaper is founded on an efficient process that integrates the modified Gerchberg-Saxton algorithm and the adjoint method. Experimental characterization of the fabricated device on a single crystal $(\overline{2} \; 01)$ gallium oxide substrate is conducted at a wavelength of 532 nm, confirming its ability to transform an incident Gaussian beam into a focused square flat-top beam. Such a device holds significant promise for various high-power laser applications, notably in laser welding and similar domains. Furthermore, because of the ultrawide bandgap of gallium oxide, DOEs operating at shorter wavelengths in the UV are also possible based on this technique.

## Introduction

Diffractive optical elements (DOEs) can precisely manipulate incident light through diffraction and play a significant role in optics. Unlike conventional optical elements reliant on refraction and reflection, DOEs feature intricately patterned microstructures on thin and lightweight substrates. These patterned microstructures deftly diffract incident light and direct it as desired, thus allowing diverse functionalities, including beam shaping. DOEs can be fabricated from a variety of materials, including fused silica^1^, sapphire^2^, diamond^3^, polymer^4^, liquid crystal^5^, and among others. They can operate across a wide range of wavelengths, such as visible^6–8^, infrared^9,10^, and terahertz^11–13^spectra, depending on their designs and applications. Such attributes render DOEs as versatile and efficient solutions across myriad applications, including imaging^14–16^, holograms^17^, laser welding^18^, and telescopes^19^.

Single-crystal gallium oxide (Ga_2_O_3_) has emerged as a promising material in the realm of semiconductors and optoelectronics. With a large bandgap of approximately 4.8 eV^20^and high breakdown electric field strength of 8 MV/cm^21^, Ga_2_O_3_ exhibits excellent potential for various electronic applications. Typically crystallizing in the monoclinic structure with several polymorphs, β-Ga_2_O_3_stands out as the most extensively researched variant due to its sizable bandgap and thermal stability^22^. This crystalline form of Ga_2_O_3 _holds great promise for optoelectronic and power electronic devices^23–25^, such as light-emitting diodes^26^, photodetectors^27,28^, power diodes^29^, and power transistors^20,30,31^. Its distinctive properties mark it as noteworthy in the realm of optics, opening new avenues for optical exploration and advancement.

Applications utilizing single crystal β-Ga_2_O_3_elements for optical applications have rarely been demonstrated, to the best of our knowledge, with existing literature focusing on the determination of the laser damage threshold, which is amongst the largest in transparent conductive oxides^32,33^, as well as on harnessing this property to realize laser accelerator nanostructures based on gratings^34^. In this work, we experimentally demonstrate a transmissive binary phase-only DOE with single-crystal gallium oxide for beam shaping. The gallium oxide utilized in this work is a commercially available $(\overline{2} \; 01)$ iron doped single crystal β-Ga_2_O_3_ substrate with dimensions of 10 mm x 10 mm x 0.62 mm. The binary phase distribution of the DOE is optimized with an efficient algorithm that integrates the modified Gerchberg-Saxton algorithm and the adjoint method. The optimized phase pattern is transferred to the gallium oxide substrate through dry etching with nickel as a hard mask to achieve the desired etch depth for π phase shift. Experimental characterization of the fabricated device confirms its shaping ability from a Gaussian beam to a square flat-top beam, which shows high potential in high-power laser applications such as welding and machining.

## Design and simulation

**Fig. 1:**
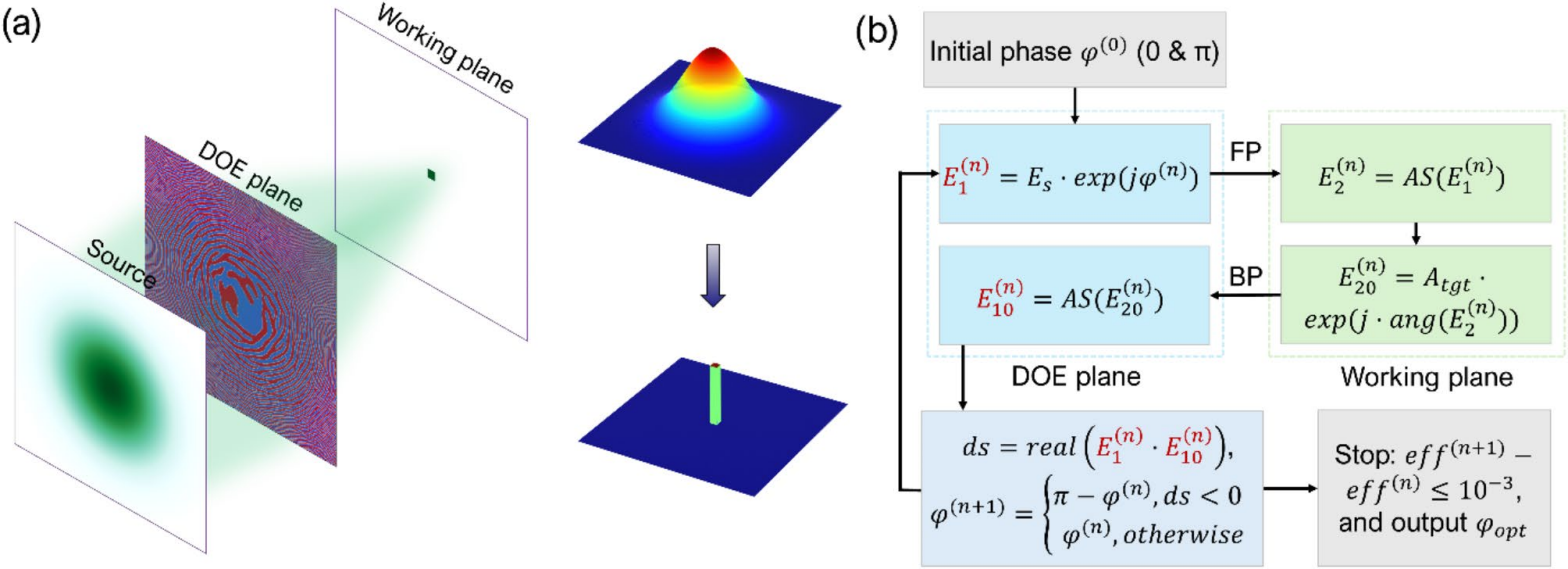
(a) Schematic of the binary DOE converting Gaussian incident wave to the square flat-top. (b) Flow chart of the proposed adjoint assisted GS algorithm.

The binary phase-only gallium oxide diffractive optical element (DOE) is designed to transform an incident beam to a desired intensity distribution. The phase distribution of the DOE is efficiently inverse-optimized by the modified Gerchberg-Saxton (GS) algorithm^35^with adjoint method^36^, as shown in flow chart in Fig. 1(a). To the best of our knowledge, such adjoint assisted GS algorithm for efficient binary phase optimization has not been proposed previously in the literature. With such a method, fewer iterations are needed compared to traditional direct binary search methods or others. Three examples are provided to demonstrate the efficiency of the proposed method. In these examples, the DOE region has binary phase distribution, which is 0 and π, consisting of 2000 × 2000 pixels with each pixel size 2.5 μm x 2.5 μm, leading to a total DOE dimension of 5 mm x 5 mm. The incident beam to the DOE is from a green laser with a central wavelength of 532 nm and Gaussian intensity distribution. In the optical measurement setup, the beam is cleaned by a spatial filter and expanded with a measured beam diameter of 4.2 mm at 1/$\:{e}^{2}$. The DOE is inverse optimized to convert the Gaussian incident beam to a focused square flat-top beam or to any other desired diffraction pattern. In the given examples, the working distance is set to be 25 mm.

As shown in Fig. 1(b), the initial binary phase distribution $\:{\phi\:}^{\left(0\right)}$ of DOE was randomly generated to have a phase of 0 or $\:\pi\:$. The modulated incident wave after passing through the DOE is $\:{E}_{1}^{\left(n\right)}={E}_{s}\bullet\:\text{e}\text{x}\text{p}\left(j{\phi\:}^{\left(n\right)}\right)$, where $\:{E}_{s}$ is the electric field distribution of the incident wave with Gaussian distribution, and $\:j=\sqrt{-1}$. The diffracted field at the working plane is obtained by the angular spectrum diffraction theory^37^, $\:{E}_{2}^{\left(n\right)}=AS\left({E}_{1}^{\left(n\right)}\right)={\mathcal{F}}^{-1}\left\{\mathcal{F}\left({E}_{1}^{\left(n\right)}\right)\bullet\:{H}_{fw}\right\},$ where $\:\mathcal{F}$ and $\:{\mathcal{F}}^{-1}$ are the 2D fast Fourier transform and its inverse. $\:{H}_{fw}=\text{e}\text{x}\text{p}(j2\pi\:z/\lambda\:\sqrt{1-{\left(\lambda\:{f}_{x}\right)}^{2}-{\left(\lambda\:{f}_{y}\right)}^{2}})$ is the forward propagation angular spectrum transfer function, where $\:z$ is the propagation distance, and $\:{f}_{x}$ and $\:{f}_{y}$ are the spatial frequency components in the and directions, respectively. The modified electric field $\:{E}_{20}^{\left(n\right)}$ on the working plane is obtained by replacing the amplitude of $\:{E}_{2}^{\left(n\right)}$ with target amplitude $\:{A}_{tgt}$ (e.g. square flat-top beam), while the phase of $\:{E}_{2}^{\left(n\right)}$ is maintained. The target magnitude $\:\left|{A}_{tgt}\right|$ is scaled by the total source irradiance divided by the total unit target irradiance. The modified electric field is propagated back to the DOE plane, $\:{E}_{10}^{\left(n\right)}=AS\left({E}_{20}^{\left(n\right)}\right)={\mathcal{F}}^{-1}\left\{\mathcal{F}\left({E}_{20}^{\left(n\right)}\right)\bullet\:{H}_{bw}\right\}$, where $\:{H}_{bw}=\text{e}\text{x}\text{p}\left(j2\pi\:\right(-z)/\lambda\:\sqrt{1-{\left(\lambda\:{f}_{x}\right)}^{2}-{\left(\lambda\:{f}_{y}\right)}^{2}})$ is the backward propagation angular spectrum transfer function. The sensitivity $\:ds={real(E}_{1}^{\left(n\right)}\bullet\:{E}_{10}^{\left(n\right)})$ is calculated to determine which pixels need to be toggled. All the pixels with $\:ds<0$ are selected and toggled to the opposite phase, that is:

$$\:{\phi\:}^{(n+1)}=\left\{\begin{array}{c}{\pi\:-\phi\:}^{\left(n\right)},\:\:\:\:\:\:\:\:\:\:\:\:\:\:ds<0\:\:\\\:{\phi\:}^{\left(n\right)},\:\:\:\:\:\:\:\:\:\:\:\:\:\:\:\:\:\:otherwise\end{array}\right.$$

Physically, $\:ds$ quantifies the effect of toggling a pixel’s phase on the overall intensity distribution at the target plane. When $\:ds<0$, it indicates that toggling the phase of the corresponding pixel to the opposite state will reduce the cost function and thus improve the system’s performance. The updated phase $\:{\phi\:}^{(n+1)}$ is passed to the new $\:{E}_{1}^{(n+1)}$ and the optimization runs iteratively. The termination criterion is set so that the conversion efficiency change $\:\left|{eff}^{(n+1)}-{eff}^{\left(n\right)}\right|\le\:{10}^{-3}$, and once this condition is met, the optimized phase distribution $\:{{\upphi\:}}_{\text{o}\text{p}\text{t}}$ is outputted. The conversion efficiency is defined as the total power inside the desired square flat-top profile divided by the total incident power.

**Fig. 2:**
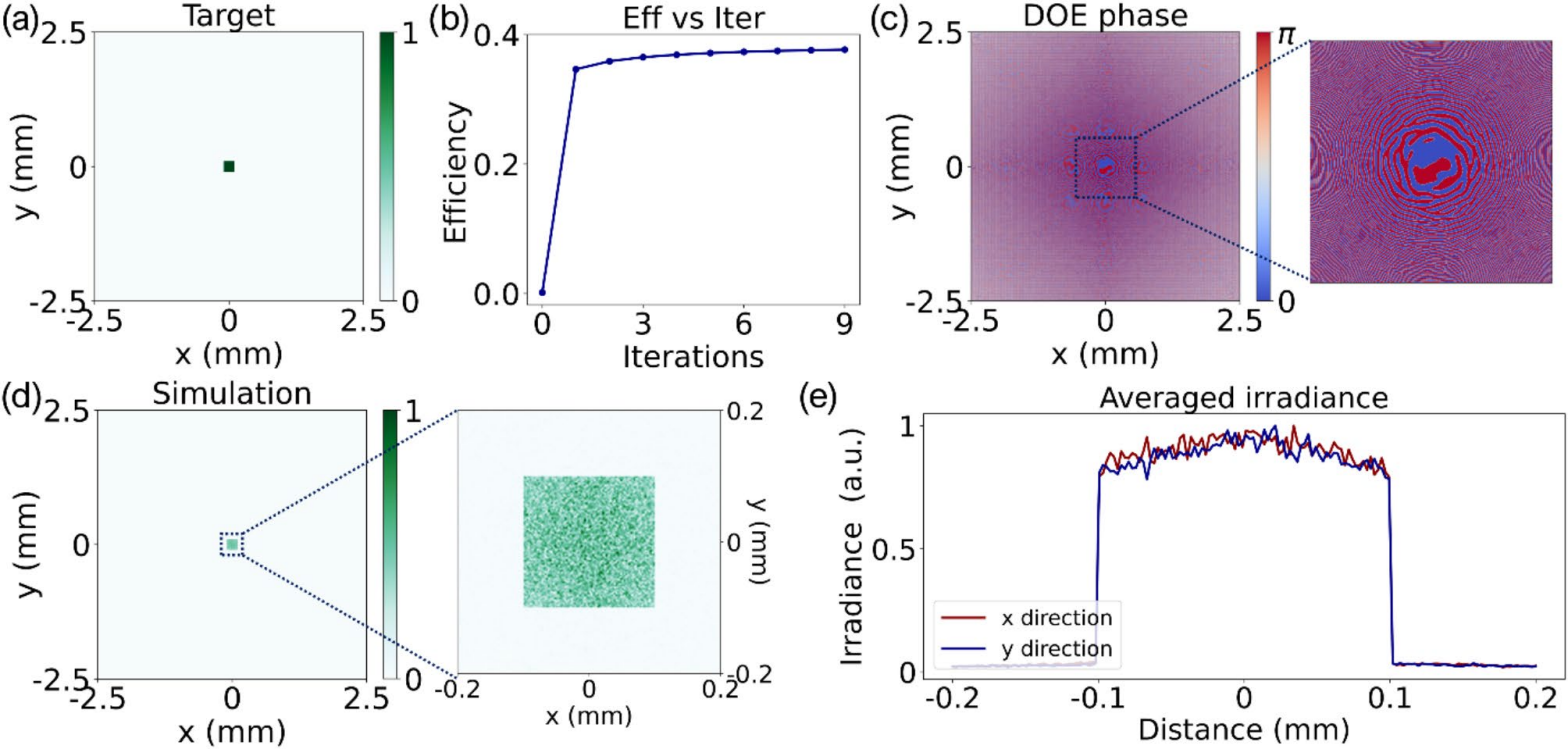
Simulation results with square flat-top target: (a) target diffractive pattern, (b) iterations vs. efficiencies, (c) optimized phase distribution of the binary DOE (blue denotes 0, red denotes π), (d) simulated diffraction pattern at working plane, and (e) averaged irradiance profiles of the diffraction pattern.

In Fig. 2(a), the target diffraction pattern is a focused square flat-top with dimensions of 200 μm x 200 μm. Through the implementation of the proposed algorithm and after 9 iterations, the conversion efficiency reaches 37.7%, as depicted in Fig. 2(b). The optimized phase distribution of the binary DOE is illustrated in Fig. 2(c), where blue indicates 0 and red denotes π. Figure 2(d) presents the normalized simulated diffraction pattern on the working plane, situated 25 mm away from the DOE plane, which closely resembles the target diffraction pattern. Upon closer inspection in the zoomed view, it is evident that the irradiance distribution is not entirely uniform, attributed to abrupt transitions between the two-phase levels. However, averaging the irradiance profiles along the x and y directions from the zoomed view of the diffraction pattern reveals a uniform distribution, as demonstrated in Fig. 2(e). This uniform distribution of irradiance is advantageous for real-world laser welding applications, where under exposure conditions, energy redistribution through thermal conduction plays a crucial role.

**Fig. 3:**
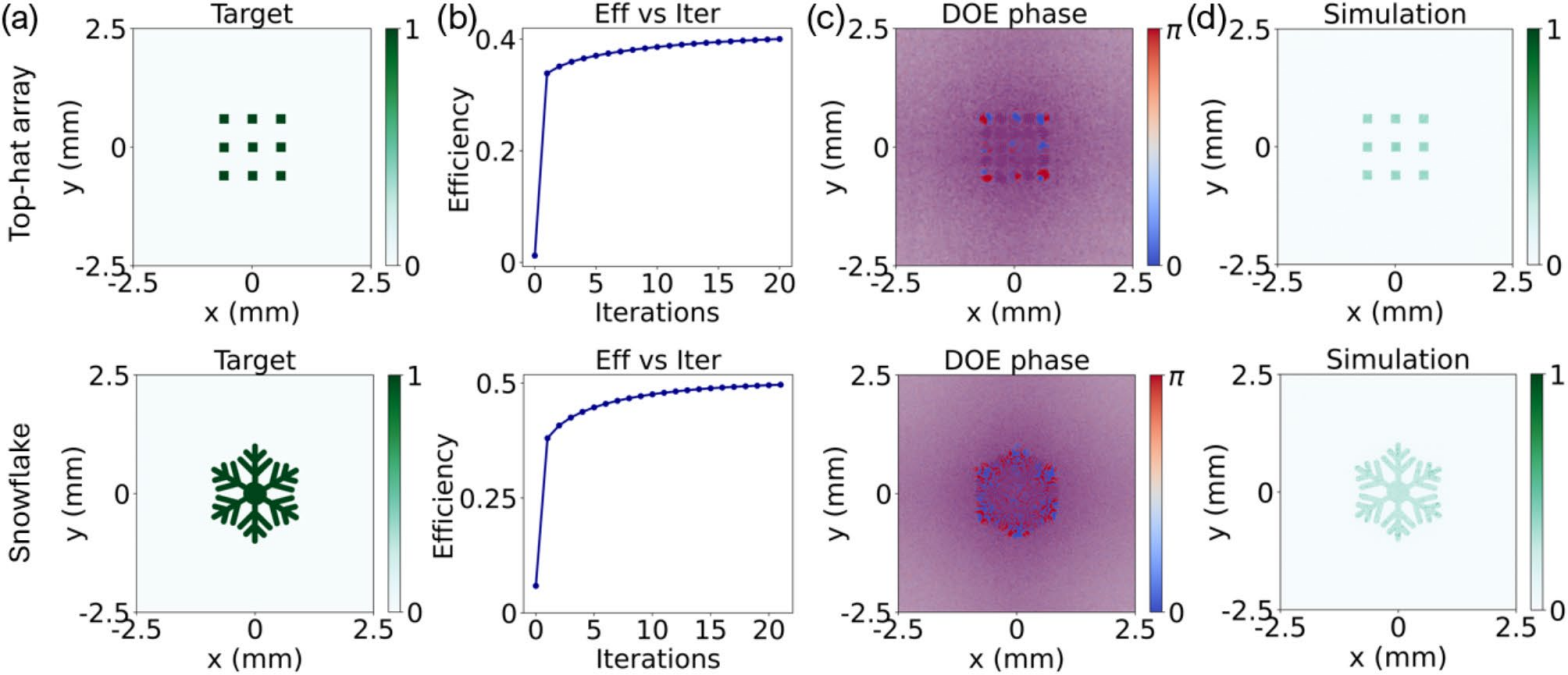
Simulation results with square flat-top array target and snowflake target: (a) target diffractive patterns, (b) iterations vs. efficiencies, (c) optimized phase distribution of the binary DOEs (blue denotes 0, red denotes π), (d) simulated diffraction pattern at the working plane.

To further demonstrate the effectiveness of the proposed adjoint-assisted GS algorithm, two additional examples featuring square flat-top array and snowflake diffraction patterns are presented, as depicted in Fig. 3(a). Figure 3(b) illustrates the evolution of efficiency with iterations. For the square flat-top array target, convergence is achieved after 20 iterations, yielding a conversion efficiency of 40.0%. Conversely, the target with a snowflake pattern requires a total of 21 iterations to reach a conversion efficiency of 49.6%. The optimized binary DOE phase distributions are displayed in Fig. 3(c). As shown in Fig. 3(d), both simulated diffraction patterns closely match the defined target patterns. During iterations, to increase the resolution in the frequency domain, the field and phase matrix are uniformly padded with zeros on each axis to form a total matrix size of 4000 × 4000. Remarkably, utilizing an Intel i7 processor, each optimization task was completed in only a few minutes, underscoring the efficiency of the proposed algorithm.

### Fabrication and measurement

To validate the designs experimentally, the square flat-top case was chosen for fabrication using a β-Ga_2_O_3_ substrate. The $(\overline{2} \; 01)$ Iron doped β-Ga_2_O_3_ substrate, purchased from Novel Crystal Technology Inc., underwent chemical mechanical polishing on both sides of the wafer and was subsequently diced into 10 mm x 10 mm pieces. The refractive index of this substrate at a wavelength of 532 nm is $\:n=1.93+j0.00$, as extracted from^38^. To achieve a π phase modulation, the pixel height is set to be $\:h=\lambda\:/2(n-1)=286$ nm, which specifies the total etched depth for the fabricated device.

**Fig. 4:**
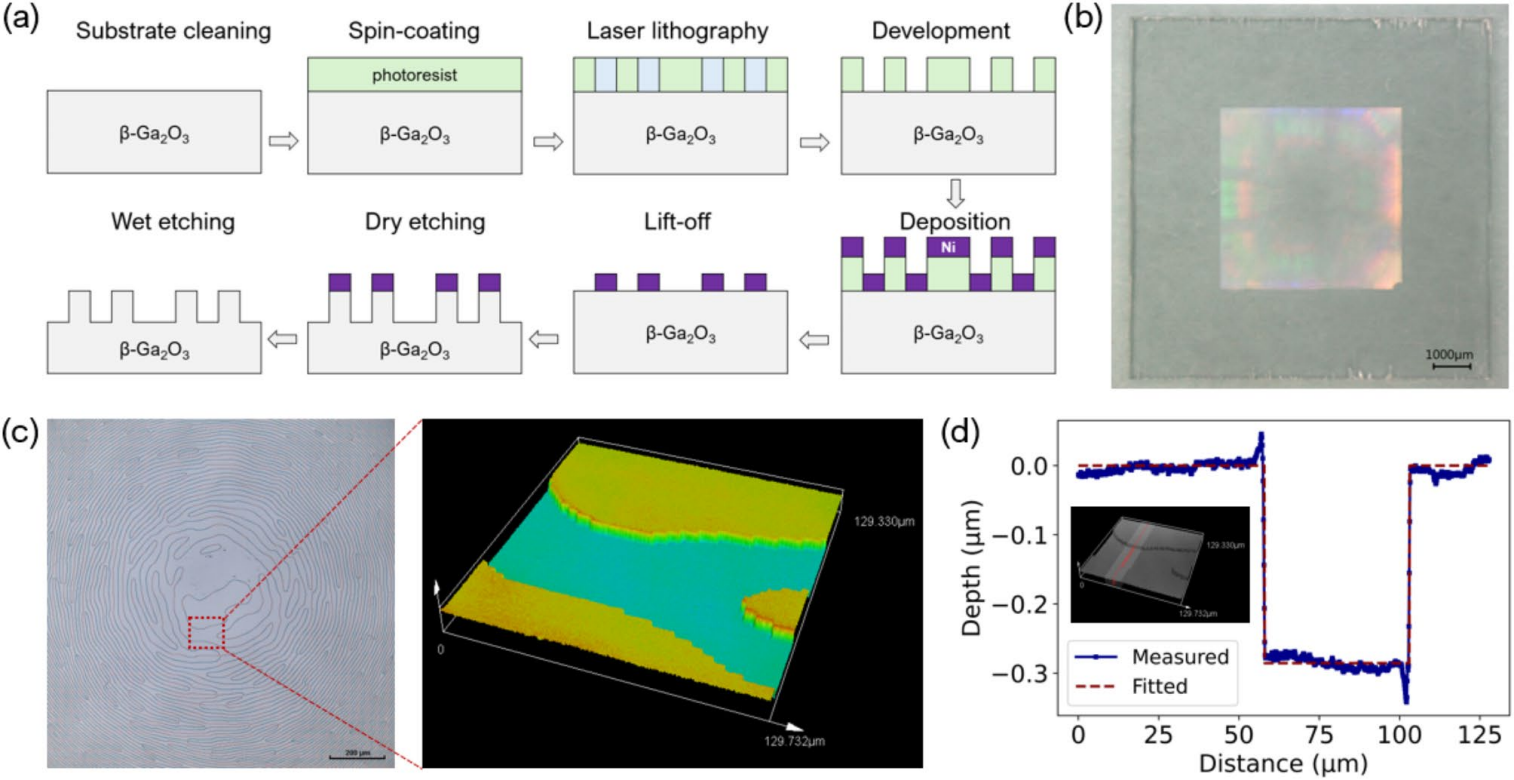
(a) Fabrication process of the binary phase β-Ga_2_O_3_ DOE. (b) Photograph of the fabricated device with a scale bar of 1000 μm (c) Microscope image of the center region of the DOE with a scale bar of 200 μm and (d) depth characterization.

The fabrication process of the β-Ga_2_O_3_ DOE with binary phase distribution is shown in Fig. 4(a). Initially, the 10 mm x 10 mm substrate underwent cleaning with piranha solution (H_2_SO_4_: H_2_O_2_ = 3: 1), followed by spin coating of positive photoresist S1813 on the substrate with Hexamethyldisilazane (HMDS) serving as an adhesion promoter and soft baking at 110 °C. Subsequently, the DWL 66 + laser lithography tool was utilized to pattern the photoresist. After development, the open windows are created on the photoresist. Next, approximately 50 nm of nickel was deposited onto the sample using an e-beam evaporation technique, followed by a lift-off process in acetone to remove the photoresist and unwanted nickel. The substrate is then introduced into the plasma dry etching tool, with forward power set at 150 W, inductively coupled plasma power at 500 W, pressure maintained at 12 mTorr, and Ar and SF_6_ flow rates at 10 sccm and 70 sccm, respectively. These conditions result in an etch rate of β-Ga_2_O_3_ of ~ 7 nm/min, with a selectivity to nickel of approximately 8.4. The total etched depth is characterized by the 3D laser scanning microscope Olympus LEXT OLS5000, as shown in Fig. 4(c). And the characterized depth is ~ 285 nm, as shown in Fig. 4(d). The remaining nickel mask on the sample was wet etched by utilizing the piranha solution. Figure 4(b) is the photograph of the fabricated device.

**Fig. 5:**
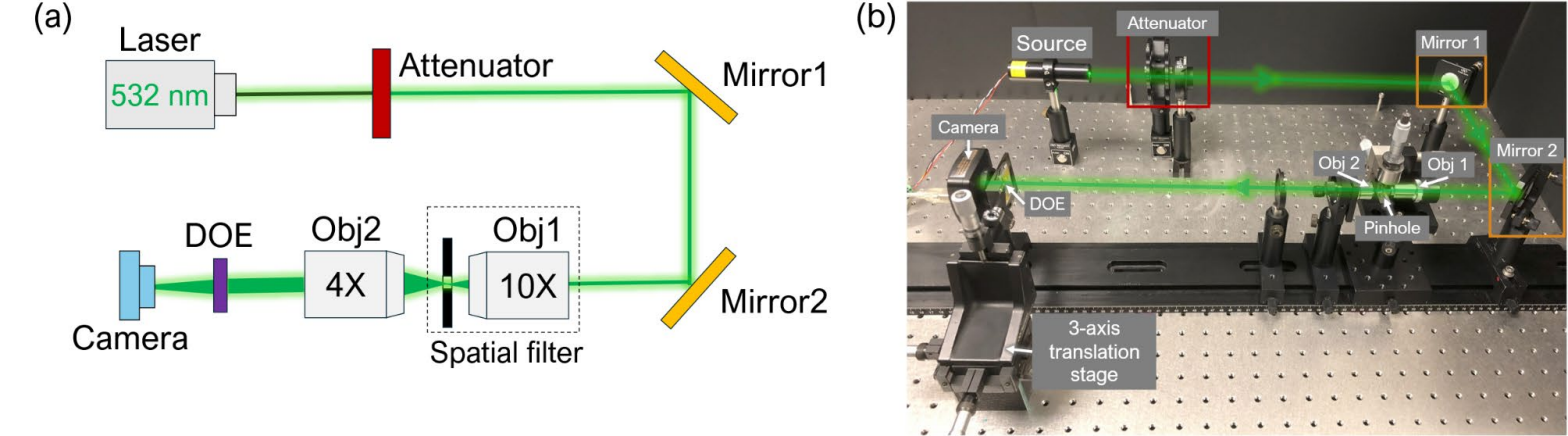
(a) Schematic of the optical measurement setup. (b) Photograph of the actual setup with drawn green lines to indicate the laser path.

The schematic of the measurement setup for characterizing the optical performance of the fabricated device is presented in Fig. 5(a), while a photograph of the actual setup is shown in Fig. 5(b). The setup comprises a 532 nm laser source, which passes through an attenuator to reduce the total laser intensity to avoid camera saturation. Two flat mirrors are employed to fold the setup and enable fine adjustment of the laser direction. To eliminate coherent artifacts on the laser beam, a spatial filter is integrated. This spatial filter incorporates a 10X objective, a 10 μm pinhole aperture, and a precision positioning mechanism. The laser beam is expanded and collimated by passing through the spatial filter and a 4X objective. The measured diameter at $\:1/{e}^{2}$ of the incident Gaussian beam to the DOE is 4.2 mm. After passing through the fabricated β-Ga_2_O_3_ DOE, the shaped beam is captured by a digital camera (MU900 from Amscope) without a lens. The camera features a resolution of 3488 × 2616 and a pixel size of 1.67 μm x 1.67 μm. To align the fabricated device into the optical path, it is positioned between the camera and the 4X objective using a three-axis translation stage.

## Results and discussion

**Fig. 6:**
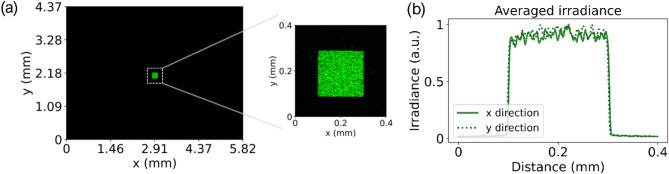
(a) Measured irradiance distribution at the working plane. (b) Averaged irradiance profiles of the measured irradiance distribution across the green channel.

We utilized a three-axis translation stage to mount the fabricated β-Ga_2_O_3_ DOE into the optical path and align it 25 mm away from the camera. The camera gain was adjusted to zero to minimize the noise. The exposure time was set to be the minimum to prevent overexposing the captured images. The measured irradiance distribution at the working plane of the fabricated DOE is shown in Fig. 6(a). From the zoomed view of Fig. 6(a), the square flat-top irradiance distribution is clearly observed, which confirms the beam shaping ability of the fabricated β-Ga_2_O_3_ DOE. The green channel averaged irradiance distributions of the square flat-top beam along both x and y directions are shown in Fig. 6(b), which reveals that the irradiance profile along each dimension is relatively uniform.

The efficiency of the fabricated β-Ga_2_O_3_ DOE was measured by first measuring the Gaussian beam irradiance distribution without the DOE mounted, followed by measuring the shaped square flat-top irradiance distribution with DOE mounted. During the measurements, the gain and exposure time of the camera are set to be the minimum. The measured efficiency is 28.1%, which is the ratio of the measured integrated irradiance across the desired square flat-top area and the integrated incident Gaussian beam irradiance. The difference between simulated and measured efficiency can be mainly attributed to the reflection loss. Due to the high refractive index of the β-Ga_2_O_3_ substrate, its reflectance at the working wavelength 532 nm is $\:{\left[({n}_{air}-{n}_{{\upbeta\:}-{Ga}_{2}{O}_{3})}\:/\:({n}_{air}+{n}_{{\upbeta\:}-{Ga}_{2}{O}_{3}})\right]}^{2}=10.1\%$. The transmitted power after the two surfaces’ reflections is 80.9%, which closely matches the transmitted power characterization of a reference β-Ga_2_O_3_ sample without any pattern. Correcting the measured efficiency for the reflection loss gives an efficiency of 34.8%, which is very close to the simulated value of 37.7%. In practice, such high reflection loss can be compensated by an anti-reflection coating of MgF_2_ on the β-Ga_2_O_3_ surface, which remains for future work.

**Fig. 7:**
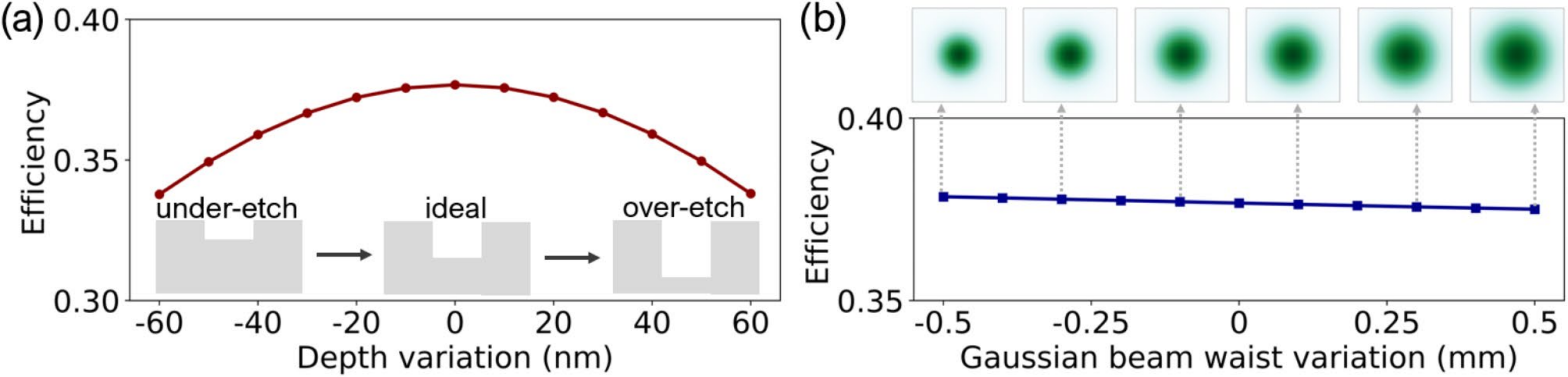
(a) Effect of etching depth variations on conversion efficiency. (b) Impact of Gaussian beam waist variations on conversion efficiency.

Fabrication imperfections and variations in the incident Gaussian beam waist also contribute to reduced efficiency, although this impact is less pronounced compared to reflection loss. In a numerical study focused on these factors, excluding reflection loss, Fig. 7 illustrates the influence of etching depth variations and Gaussian beam waist fluctuations on the DOE conversion efficiency. As depicted in Fig. 7(a), at the ideal etching depth of 286 nm (0 nm variation), the efficiency peaks at 37.7%. However, deviations from this ideal, whether over-etched or under-etched, result in a reduced efficiency of 33.8%, marking a ~ 10% efficiency decline with a phase variation of 0.2π. In Fig. 7(b), a linear relationship between Gaussian beam waist variation and efficiency is depicted. Overall, as the waist variation varies in ± 24%, the device efficiency shows minimal variation, remaining nearly constant.

**Fig. 8:**
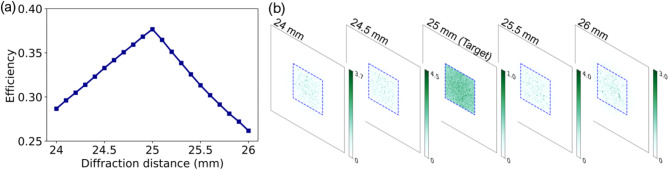
(a) Effect of diffraction distance on conversion efficiency. (b) Diffraction irradiance distributions on planes at varied distances from the DOE.

The impact of diffraction distance on beam conversion efficiency is illustrated in Fig. 8. The device is optimized with a single working plane situated 25 mm away, where it exhibits peak efficiency. Deviating from this optimal distance by ± 4% results in a linear decrease in conversion efficiency, as depicted in Fig. 8(a). Specifically, at a diffraction distance of 24 mm, efficiency declines to 28.7%, whereas at 26 mm, it drops to 26.2%. Notably, longer diffraction distances show a more pronounced efficiency drop compared to shorter ones. Within the range of ± 1.2%, the device maintains an efficiency above 35%. The irradiance distributions across the various diffraction planes are visualized in Fig. 8(b). Each distribution is normalized relative to the maximum irradiance at a 25 mm diffraction distance, and the desired square flat-top region is represented by a dashed blue square measuring 200 μm x 200 μm. Observation reveals that at 25 mm, the beam exhibits a relatively uniform intensity distribution with a square flat-top. Moving away from this optimal plane results in a degradation of beam uniformity.

## Conclusion

In conclusion, this study introduces an efficient optimization approach for designing binary phase-only diffractive optical elements. The method integrates the modified Gerchberg-Saxton algorithm and adjoint method, leading to a significant acceleration of the design process. The designed DOE is fabricated on $(\overline{2} \; 01)$ iron-doped β-Ga_2_O_3_ via a dry etching method. The DOE effectively transforms a Gaussian incident beam into a focused square flat-top distribution at a wavelength of 532 nm, offering potential applications in high-power beam shaping. Looking forward, the next step involves exploring the fabrication of multilevel phase-only β-Ga_2_O_3_ DOEs through grayscale lithography and etching processes.

## Data Availability

Data underlying the results presented in this paper are not publicly available at this time but may be obtained from the corresponding authors upon reasonable request.

## References

[1] Britten, J. A. & Summers, L. J. Multiscale, multifunction diffractive structures wet etched into fused silica for high-laser damage threshold applications. *Appl. Opt.* **37**, 7049 (1998). 18301523 10.1364/ao.37.007049

[2] Li, Q. K. et al. Multilevel phase-type diffractive lens embedded in sapphire. *Opt. Lett.* **42**, 3832 (2017). 28957140 10.1364/OL.42.003832

[3] Wildi, T., Kiss, M. & Quack, N. Diffractive optical elements in single crystal diamond. *Opt. Lett.* **45**, 3458 (2020). 32630871 10.1364/OL.393679

[4] Wang, H., Wang, H., Zhang, W. & Yang, J. K. W. Toward near-perfect diffractive optical elements via nanoscale 3D printing. *ACS Nano*. **14**, 10452–10461 (2020). 32687316 10.1021/acsnano.0c04313

[5] Luo, Z., Li, Y., Semmen, J., Rao, Y. & Wu, S. T. Achromatic diffractive liquid-crystal optics for virtual reality displays. *Light Sci. Appl.* **12**, 230 (2023). 37714841 10.1038/s41377-023-01254-8 PMC10504380

[6] Chen, H. et al. *Diffractive Deep Neural Networks Visible Wavelengths Eng.* **7**, 1483–1491 (2021).

[7] Jia, W., Menon, R. & Sensale-Rodriguez, B. Visible and near-infrared programmable multi-level diffractive lenses with phase change material Sb2S3. *Opt. Express*. **30**, 6808–6817 (2022). 35299459 10.1364/OE.452472

[8] Xiao, X. et al. Large-scale achromatic flat lens by light frequency-domain coherence optimization. *Light Sci. Appl.* **11**, 323 (2022). 36357364 10.1038/s41377-022-01024-y PMC9649754

[9] Blough, C. G., Rossi, M., Mack, S. K. & Michaels, R. L. Single-point diamond turning and replication of visible and near-infrared diffractive optical elements. *Appl. Opt.* **36**, 4648 (1997). 18259260 10.1364/ao.36.004648

[10] Khonina, S. N. & Karpeev, S. V. Generating inhomogeneously polarized higher-order laser beams by use of diffractive optical elements. *J. Opt. Soc. Am. A*. **28**, 2115 (2011). 10.1364/JOSAA.28.002115 21979517

[11] Siemion, A. The magic of optics—an overview of recent advanced terahertz diffractive optical elements. *Sensors* **21**, 100 (2020). 33375221 10.3390/s21010100 PMC7795556

[12] Furlan, W. D. et al. 3D printed diffractive terahertz lenses. *Opt. Lett.* **41**, 1748 (2016). 27082335 10.1364/OL.41.001748

[13] Jia, W., Lou, M., Gao, W. & Sensale-Rodriguez, B. Design and fabrication of a terahertz dual-plane hologram and extended-depth-of-focus diffractive lens. *Opt. Continuum*. **1**, 1722–1729 (2022).

[14] Anand, V., Katkus, T. & Juodkazis, S. Randomly multiplexed diffractive lens and axicon for spatial and spectral imaging, Micromachines 11, 437 (2020). 10.3390/mi11040437 PMC7231349 32326337

[15] Heide, F., Fu, Q., Peng, Y. & Heidrich, W. Encoded diffractive optics for full-spectrum computational imaging. *Sci. Rep.* **6**, 33543 (2016). 27633055 10.1038/srep33543 PMC5025844

[16] Jia, W., Lin, D., Menon, R. & Sensale-Rodriguez, B. Multifocal multilevel diffractive lens by wavelength multiplexing. *Appl. Opt.* **62**, 6931 (2023). 37707032 10.1364/AO.497775

[17] Jia, W., Lin, D. & Sensale-Rodriguez, B. Machine learning enables multi‐degree‐of‐freedom reconfigurable terahertz holograms with cascaded diffractive optical elements. *Adv. Opt. Mater.* **11**, 2202538 (2023).

[18] Kang, S. & Shin, J. Laser beam oscillation welding of aluminum alloy using the spatially modulated beam by diffractive optical element (DOE). *J. Manuf. Process.* **66**, 387–396 (2021).

[19] Zhang, H., Liu, H., Xu, W. & Lu, Z. Large aperture diffractive optical telescope: a review. *Opt. Laser Technol.* **130**, 106356 (2020).

[20] Higashiwaki, M., Sasaki, K., Kuramata, A., Masui, T. & Yamakoshi, S. Gallium oxide (Ga_2_O_3_) metal-semiconductor field-effect transistors on single-crystal β-Ga_2_O_3_ (010) substrates. *Appl. Phys. Lett.* **100**, 013504 (2012).

[21] Ghosh, K. & Singisetti, U. Impact ionization in β-Ga_2_O_3_. *J. Appl. Phys.* **124**, 085707 (2018).

[22] Zhang, J., Shi, J., Qi, D. C., Chen, L. & Zhang, K. H. L. Recent progress on the electronic structure, defect, and doping properties of Ga_2_O_3_. *APL Mater.* **8**, 020906 (2020).

[23] Green, A. J. et al. *β-Gallium Oxide Power Electron. APL Mater.* **10**, 029201 (2022).

[24] Kim, M., Seo, J. H., Singisetti, U. & Ma, Z. Recent advances in free-standing single crystalline wide band-gap semiconductors and their applications: GaN, SiC, ZnO, β-Ga_2_O_3_, and diamond. *J. Mater. Chem. C*. **5**, 8338–8354 (2017).

[25] Galazka, Z. β-Ga_2_O_3_ for wide-bandgap electronics and optoelectronics. *Semicond. Sci. Technol.* **33**, 113001 (2018).

[26] Huang, Y., Saito, K., Tanaka, T. & Guo, Q. Strategy toward white LEDs based on vertically integrated rare earth doped Ga_2_O_3_ films. *Appl. Phys. Lett.* **119**, 062107 (2021).

[27] Nakagomi, S., Momo, T., Takahashi, S. & Kokubun, Y. Deep ultraviolet photodiodes based on β-Ga_2_O_3_/SiC heterojunction. *Appl. Phys. Lett.* **103**, 072105 (2013).

[28] Suzuki, R., Nakagomi, S., Kokubun, Y., Arai, N. & Ohira, S. Enhancement of responsivity in solar-blind β-Ga_2_O_3_ photodiodes with a au Schottky contact fabricated on single crystal substrates by annealing. *Appl. Phys. Lett.* **94**, 222102 (2009).

[29] Xue, H. et al. An overview of the ultrawide bandgap Ga_2_O_3_ semiconductor-based schottky barrier diode for power electronics application. *Nanoscale Res. Lett.* **13**, 290 (2018). 30232628 10.1186/s11671-018-2712-1 PMC6145968

[30] Higashiwaki, M. et al. Depletion-mode Ga_2_O_3_ metal-oxide-semiconductor field-effect transistors on β-Ga_2_O_3_ (010) substrates and temperature dependence of their device characteristics. *Appl. Phys. Lett.* **103**, 123511 (2013).

[31] Wong, M. H. & Higashiwaki, M. Vertical β-Ga_2_O_3_ power transistors: a review. *IEEE Trans. Electron. Devices*. **67**, 3925–3937 (2020).

[32] Yoo, J. H., Rafique, S., Lange, A., Zhao, H. & Elhadj, S. Lifetime laser damage performance of β-Ga2O3 for high power applications. *APL Mater.* **6**, 036105 (2018).

[33] Yoo, J. H., Lange, A., Chesser, J., Falabella, S. & Elhadj, S. A Survey of Transparent conducting films and Optoelectrical materials for high Optical Power Applications. *Phys. Status Solidi (a)*. **216**, 1900459 (2019).

[34] Deng, H. et al. Gallium Oxide for High-Power Optical Applications. *Adv. Opt. Mater.* **8**, 1901522 (2020).

[35] Gerchber, R. W. & Saxton, W. O. A practical algorithm for the determination of the phase from image and diffraction plane pictures. *Optik* **35**, 237 (1972).

[36] Lalau-Keraly, C. M., Bhargava, S., Miller, O. D. & Yablonovitch, E. *Adjoint Shape Optim. Appl. Electromagn. Des. Opt. Express* **21**, 21693 (2013). 10.1364/OE.21.021693 24104043

[37] Goodman, J. W. *Introduction to Fourier Optics* 3rd edn (Roberts & Co, 2005).

[38] Onuma, T. et al. Spectroscopic ellipsometry studies on β-Ga_2_O_3_ films and single crystal. *Jpn J. Appl. Phys.* **55**, 1202B2 (2016).

